# Oxidant-Antioxidant Balance during On-Pump Coronary Artery Bypass Grafting

**DOI:** 10.1155/2014/263058

**Published:** 2014-08-18

**Authors:** Umit Mentese, Orhan Veli Dogan, Ibrahim Turan, Sefer Usta, Emre Dogan, Seda Oztas Mentese, Selim Demir, Tanil Ozer, Ahmet Cagri Aykan, Ahmet Alver

**Affiliations:** ^1^Department of Cardiovascular Surgery, Ahi Evren Thoracic and Cardiovascular Surgery Training and Research Hospital, 61040 Trabzon, Turkey; ^2^Department of Cardiovascular Surgery, Faculty of Medicine, Sakarya University, 54180 Sakarya, Turkey; ^3^Department of Genetic and Bioengineering, Faculty of Engineering and Natural Sciences, Gumushane University, 29100 Gumushane, Turkey; ^4^Department of Emergency Medicine, Kanuni Training and Research Hospital, 61290 Trabzon, Turkey; ^5^Department of Medical Biochemistry, Faculty of Medicine, Karadeniz Technical University, 61080 Trabzon, Turkey; ^6^Department of Cardiology, Ahi Evren Thoracic and Cardiovascular Surgery Training and Research Hospital, 61040 Trabzon, Turkey

## Abstract

*Backround*. The aim of this study was to evaluate the changes in perioperative oxidant-antioxidant balance in ONCABG. *Methods*. Twenty-three patients were included in this study. Serum total oxidant status (TOS), total antioxidant status (TAS), and oxidative stress index (OSI) values were assessed preoperatively, at 20 minutes after aortic clamping and at 30 minutes, 6 hours, and 48 hours after declamping (reperfusion). The patients were divided into 2 groups according to the median aortic cross clamping (XC) time: group 1 (XC time < 42 minutes) and group 2 (XC time ≥ 42 minutes). * Results*. TOS and OSI values of whole patients at 30 minutes after reperfusion were higher than preoperative values (*P* = 0.045, *P* = 0.015), while perioperative TAS levels of the patients were similar to the preoperative levels (*P* = 0.173). XC time was correlated with TOS levels at 30 minutes after reperfusion (*r* = 0.43, *P* = 0.041). In group 2, TOS and OSI values at 30 minutes after reperfusion were higher than preoperative values (*P* = 0.023, *P* = 0.048), whereas a significant difference was not found in group 1 (*P* = 0.601, *P* = 0.327). * Conclusions*. Oxidative imbalance and increase in TOS at reperfusion in ONCABG may be associated with XC time.

## 1. Introduction

Coronary artery bypass grafting (CABG) induces oxidative stress (OS). This situation is closely associated with overproduction of reactive oxygen species (ROS). ROS plays an important role in the physiopathogenesis of OS [1–4]. ROS have toxic effects on cell structures including lipids, proteins, and nucleic acids. The oxidative reaction leads injury to cell function and may enhance the perioperative or postoperative complications after CABG [5, 6].

However, ROS that have been considered not only as lethal byproducts of cellular metabolism but also as important molecules in vascular signaling are normally present in cells. The production of ROS in the organisms and their degradation by antioxidants is in balance. When ROS exceeds the antioxidant capacity, this situation results in OS [3, 5, 7]. Thus, evaluation of oxidants and antioxidants together in patients undergoing CABG may yield a more objective view about the change in the oxidative balance.

Serum concentrations of different oxidative components can be measured in the laboratory separately, but the measurement of these molecules is labor intensive and requires much time, complicated techniques, and cost. Total antioxidant status (TAS), total oxidant status (TOS), and oxidative stress index (OSI) reflect the redox balance between oxidation and antioxidation. TAS measurement is an indicatorof the activity of all antioxidants while TOS is anindicator of ROS. OSI is the ratio of TOS to TAS and an indicator of OS degree [8, 9].

There are various obstacles to devise appropriate strategies for optimal perioperative antioxidant therapy. Contribution of the various mechanisms on the oxidant-antioxidant balance during on-pump coronary artery bypass grafting (ONCABG) has not been fully evaluated yet [2, 4]. In this study, we have evaluated the changes in oxidant-antioxidant balance and its relation to XC time by measuring TOS, TAS and calculating OSI in ONCABG patients.

## 2. Materials and Methods

### 2.1. Study Population

A total of 23 patients (17 males, 6 females) who underwent elective isolated ONCABG were included in this study. Patients with any of the following criteria were excluded: acute coronary syndrome, emergency surgery, reoperation, combined procedures (e.g., valvular surgery), off-pump coronary artery bypass grafting (OPCAB), as well as patients with chronic inflammatory disease (such as rheumatoid arthritis, malignancy, or psoriasis), with autoimmune disease, or receiving immunosuppressive drugs. None of the patients used vitamins or dietary supplements. Informed consent was obtained from all patients, and approval for the study was given by the local ethics committee (2013-164).

The median aortic cross clamping (XC) time was 42 minutes. We divided the patients into 2 groups according to median XC time. Group 1 consists of 11 patients (8 males, 3 females) with a below median XC time and group 2 consists of 12 patients (9 males, 3 females) with a median and above median XC time.

### 2.2. Surgical Technique

The same surgical and anesthetic team managed all patients. Patients received premedication with midazolam. Anesthesia was induced with midazolam, fentanyl, thiopental, and rocuronium, while sevoflurane and fentanyl were used as maintenance. Radial and pulmonary arterial catheters were introduced under local anesthesia. After standard general anesthesia, a median sternotomy was performed, followed by routine aortic and right atrial cannulation. Cardiopulmonary bypass (CPB) was carried out using membrane oxygenators and moderate systemic hypothermia. Myocardial protection was achieved by antegrade mild hypothermic (32°C) blood cardioplegia, repeated every 20 min. Heparin 3.0 mg/kg was administered, and the activated clotting time was maintained >400 sec during the procedure. Heparin was neutralized with protamine in a ratio of 1 : 1.3 within 10 minutes after the end of CPB. After surgery, all patients were followed up in the intensive care unit.

### 2.3. Sample Collection

Blood samples for TOS and TAS determination were drawn preoperatively, 20 minutes after aortic cross clamping and just before the administration of the second cardioplegia (AC 20 min), and 30 minutes (Rp 30 min), 6 hours (Rp 6 h), and 48 hours (Rp 48 h) after cross clamp release (reperfusion) from the arterial line. The blood samples were kept at room temperature for 30 minutes and then separated from the cells by centrifugation at 3000 rpm for 5 minutes. Serum samples were stored at −80°C until the day of biochemical analysis.

### 2.4. Biochemical Assays

#### 2.4.1. Measurement of Total Oxidant Status (TOS)

TOS levels were determined using a method previously described by Erel [9] and calculated in *μ*mol H_2_O_2_ equivalent/L.

#### 2.4.2. Measurement of Total Antioxidant Status (TAS)

TAS levels were determined using a method developed by Erel [10] and calculated in mmol Trolox equivalent/L.

#### 2.4.3. Calculation of Oxidative Stress Index (OSI)

The TOS : TAS ratio was used as OSI. To perform the calculation, the unit of TAS, mmol Trolox equivalent/L, was converted to *μ*mol Trolox equivalent/L, and OSI was calculated as follows: OSI = [(TOS, *μ*mol H_2_O_2_ equivalent/L)/(TAS, *μ*mol Trolox equivalent/L) × 100].

### 2.5. Statistical Analysis

Descriptive statistics for the studied variables (characteristics) were presented as Mean ± Standard Deviation. Two-factor experiment with repeated measures on one factor analysis of variance was used to compare the groups and periods for TAS, TOS, and OSI. Following analysis of variance, Tukey's multiple comparison test was performed to determine different periods. In addition, Student's* t*-test was executed to compare groups in terms of other characteristics. For determining the relationships between the characteristics, Spearman's correlation coefficient was calculated, and for determining the relationships between groups and categorical variables, chi-square test was performed. Statistical significance levels were considered as *P* < 0.05 and SPSS (version 13) statistical program was used for all statistical computations.

## 3. Results

Groups were similar in terms of age, gender, and BSA. The demographic characteristics of the patients are presented in Table 1. The mean cross clamping time was 44.87 ± 19.21 minutes (29.18 ± 9.5 minutes in group 1 and 59.25 ± 13.58 minutes in group 2). There was no in-hospital mortality, severe cardiopulmonary, or vascular morbidity. Perioperative variables of the patients are presented in Table 2.

When all cases of ONCABG were evaluated, the TOS and OSI values of Rp 30 min were significantly higher than the preoperative TOS and OSI values (*P* = 0.045, *P* = 0.015, resp.), while perioperative TAS levels of the patients were similar with the preoperative TAS levels (*P* = 0.173). There was a positive correlation between cross clamping time and TOS levels at Rp 30 min (*r* = 0.43, *P* = 0.041).

In group 2 TOS and OSI values at Rp 30 min were significantly higher than the preoperative values (*P* = 0.023, *P* = 0.048, resp.), while perioperative TOS and OSI values of group 1 were similar to the preoperative values (*P* = 0.601, *P* = 0.327, resp.). TOS, TAS, and OSI values of the patients are presented in Table 3.

## 4. Discussion

We found that the patients undergoing ONCABG are exposed to potent oxidative imbalance and increase in oxidants at reperfusion which are associated with duration of XC.

CABG procedure induces overproduction of ROS (hydroxyl, hydrogen peroxide, hypochlorite, and superoxide) which originates from various enzymatic and cellular sources [1, 3, 4]. Determining TOS is more practical than individual ROS measurements [9]. In the present study, the significant increase in TOS levels at Rp 30 min shows an increase in ROS production in ONCABG patients.

On the other hand, controversial findings were reported on the perioperative course of antioxidants in CABG patients [3, 4, 6, 11, 12]. The effects of antioxidant molecules in serum are additive. The cooperation of antioxidants in serum provides protection for the organism against ROS. The real antioxidant status of the organism may not be reflected by individual measurement of antioxidants. Measurement of TAS ought to be essential in evaluation of the real antioxidant status [8, 9, 13]. In the present study, perioperative TAS levels of patients were similar to the basal levels.

Oxidative stress is an oxidant-antioxidant imbalance status, due to oxidants which exceed the antioxidant capacity. OSI is the ratio of TOS to TAS and is an indicator of OS degree [8, 9, 13]. It may offer a more accurate comment for the evaluation of the change in oxidant-antioxidant balance. In the present study, we found significant increase of OSI values at Rp 30 min. This finding suggested the development of oxidative imbalance or increase in the degree of OS in ONCABG patients.

In ONCABG patients there are many causes of oxidative stress such as surgical damage, CPB, and ischemia reperfusion injury [4, 6, 14]. In our study, we aimed to detect a different finding in terms of assessment of oxidant-antioxidant balance, in the ischemia period (AC 20 min), and also tried to distinguish the effect of reperfusion. If blood samples for the ischemia period were taken just before aortic cross clamping was removed, the effects of ischemia as well as the effects of reperfusion due to the cardioplegias could have occurred [6]. Some of the surgical manipulations had been done and CPB had started before this part of the intervention (AC 20 min) as well. The presence of an increase in TOS and OSI at Rp 30 min, with absence of an increase at AC 20 min, suggests that reperfusion may be a major inducer of oxidative imbalance in ONCABG.

Ischemic period associated with oxidative stress in CABG. Authors reported that XC time was correlated with total peroxide, 8-isoprostane, and nitrites/nitrates levels in patients undergoing CABG [15, 16]. We also detected a positive correlation between XC time and TOS levels at Rp 30 min.

Ferrari et al. reported a significant and sustained increase in OS in 10 patients undergoing ONCABG with a mean XC duration of 55.2 minutes, while a mild and temporary increase in OS in 10 patients undergoing ONCABG with a mean XC duration of 25.2 minutes [17]. Nowicki et al. also reported the absence of a significant oxidative damage in the myocardial biopsy specimens of 8 patients undergoing ONCABG with a mean XC duration of 29.5 minutes [18]. To our knowledge, this paper is the first in the literature which reported neither systemic oxidant increase nor systemic oxidative imbalance occurred in patients with a mean XC duration of 29 minutes (group 1) by evaluating TOS and OSI. On the contrary, significant systemic oxidant increase and systemic oxidative imbalance were observed in group 2. The increase in TOS and OSI values of whole patients was mostly affected by the increase in TOS and OSI values of patients in group 2.

The oxidative balance change which was more significant at group 2 might be affected from demographic data of patients. The relation between HT and smoking with oxidative balance changes has been reported previously [19, 20]. In our study this parameters' statistical similarity might be caused by the fewness of patients. Besides, similarity of demographic characteristics of dispersed patients into groups may enable us to isolate XC time effect on TOS, TAS, and OSI from the other potential effective statuses like HT, smoking, any drug utilizations, and so forth.

The CPB duration of the groups was also different. But there was no difference in terms of aerobic pump times. Prolongation of CPB time in group 2 was mainly due to longer length of XC duration.

Clinical trials conducted with a variety of antioxidant strategies have been largely disappointing. Optimal design of antioxidant strategies in patients undergoing ONCABG needs detailed evaluation of oxidative phenomenon [2, 6]. In our study, the oxidant-antioxidant balance was not affected in ONCABG patients with a short XC duration, so we believe that widespread antioxidant therapy may not be needed. Administration of antioxidants in selected cases may be more effective. Studies to determine the critical XC durations in terms of oxidative imbalance may be designed. Further larger-scale prospective studies are required for accurate strategies to this clinical phenomenon.

The major limitation of the current study may be the relatively small number of patients.

## 5. Conclusions

In conclusion, according to assessments of TAS, TOS, and OSI, an oxidative imbalance or increase in OS degree was observed in patients who underwent ONCABG, especially due to an increase in TOS at reperfusion. Moreover, not only increase in TOS at reperfusion but also oxidative imbalance seems to be associated with XC time. These findings inspire further studies which may provide important criteria for antioxidant therapy.

## Figures and Tables

**Table 1:** Demographic data of groups.

	Whole patients (*n* = 23)	Group 1 (*n* = 11)	Group 2 (*n* = 12)	*P* value∗
Age, year	62.87 ± 10.310	63.64 ± 8.582	62.17 ± 12.021	0.741
Male, *n* (%)	17 (73.9%)	8 (72.7%)	9 (75.0%)	0.901
BSA (m^2^)	1.838 ± 0.175	1.848 ± 0.193	1.828 ± 0.165	0.793
Urea (mg/dL)	38.13 ± 10.385	35.91 ± 13.217	40.17 ± 6.887	0.338
Creatinine (mg/dL)	0.939 ± 0.282	0.918 ± 0.299	0.958 ± 0.278	0.742
ALT (U/L)	28.57 ± 18.571	22.64 ± 10.856	34.00 ± 22.700	0.147
AST (U/L)	32.96 ± 18.826	35.09 ± 22.170	31.00 ± 15.915	0.614
ACE inhibitors, *n* (%)	11 (47.8%)	4 (36.4%)	7 (58.3%)	0.292
Calcium antagonists, *n* (%)	1 (4.3%)	0 (0%)	1 (8.3%)	0.328
Beta blockers, *n* (%)	4 (17.4%)	2 (18.2%)	2 (16.7%)	0.924
Nitrates, *n* (%)	5 (21.7%)	2 (18.2%)	3 (25.0%)	0.692
HT, *n* (%)	17 (73.9%)	7 (63.6%)	10 (83.3%)	0.283
DM, *n* (%)	5 (21.7%)	2 (18.2%)	3 (25.0%)	0.692
COPD, *n* (%)	3 (13.0%)	2 (18.2%)	1 (8.3%)	0.484
PAD, *n* (%)	2 (8.7%)	1 (9.1%)	1 (8.3%)	0.949
CVD, *n* (%)	1 (4.3%)	0 (0%)	1 (8.3%)	0.328
Smoking, *n* (%)	7 (30.4%)	2 (18.2%)	5 (41.7%)	0.221
Alcohol, *n* (%)	1 (4.5%)	1 (9.1%)	0 (0%)	0.306
CCS class	2.26 ± 0.449	2.36 ± 0.505	2.17 ± 0.389	0.304
LVEF	53.48 ± 9.224	52.27 ± 8.475	54.58 ± 10.104	0.561
LMCAD	1 (4.3%)	0 (0%)	1 (8.3%)	0.328

∗Comparison of group 1 and group 2.

ACE: angiotensin converting enzyme, ALT: alanine aminotransferase, AST: aspartate aminotransferase, BSA: body surface area, CCS: Canadian Cardiovascular Society, COPD: chronic obstructive pulmonary disease, CVD: cerebrovascular disease, DM: diabetes mellitus, HT: hypertension, LMCAD: left main coronary artery disease, LVEF: left ventricular ejection fraction, and PAD: peripheral arterial disease.

**Table 2:** Perioperative variables of groups.

	Whole patients (*n* = 23)	Group 1 (*n* = 11)	Group 2 (*n* = 12)	*P* value∗
Number of grafts	2.913 ± 0.996	2.636 ± 0.809	3.167 ± 1.115	0.209
LIMA use, *n* (%)	20 (87%)	10 (90.9%)	10 (83.3%)	0.590
ACC time (min)	44.87 ± 19.212	29.18 ± 9.506	59.25 ± 13.579	0.001
CPB time (min)	98.43 ± 32.481	75.64 ± 21.463	119.33 ± 26.362	0.001
Aerobic pump time (min)	53.57 ± 20.273	46.45 ± 20.447	60.08 ± 18.574	0.109
Ventilation time (h)	8.22 ± 2.864	7.86 ± 1.951	8.54 ± 3.564	0.583
ICU stay (h)	31.87 ± 15.256	28.64 ± 15.826	34.83 ± 14.758	0.342
Hospital stay (day)	5.61 ± 0.783	5.64 ± 0.924	5.58 ± 0.669	0.875

∗Comparison of group 1 and group 2.

ACC: aortic crossclamp, CPB: cardiopulmonary bypass, h: hours, ICU: intensive care unit, LIMA: left internal mammary artery, and min: minutes.

**Table 3:** TOS, TAS, and OSI values of groups.

	Whole patients (*n* = 23)	Group 1 (*n* = 11)	Group 2 (*n* = 12)
TOS levels (*μ*mol H_2_O_2_ equivalent/L.)			
Preoperative	10.14 ± 7.68	9.53 ± 8.08	10.69 ± 7.65
AC 20 min	8.81 ± 3.57	8.88 ± 4.20	8.76 ± 3.23
Rp 30 min	12.47 ± 6.41^a^	9.83 ± 3.10	15.12 ± 7.82^d^
Rp 6 h	11.72 ± 6.60	9.06 ± 3.35	14.16 ± 7.96^e^
Rp 48 h	8.79 ± 2.83	7.86 ± 1.55	10.08 ± 3.73
TAS levels (mmol Trolox equivalent/L)			
Preoperative	1.91 ± 0.31	1.98 ± 0.37	1.86 ± 0.25
AC 20 min	1.83 ± 0.24	1.83 ± 0.20	1.82 ± 0.29
Rp 30 min	1.79 ± 0.26	1.74 ± 0.26^c^	1.83 ± 0.27
Rp 6 h	1.92 ± 0.28	1.82 ± 0.21	2.01 ± 0.31
Rp 48 h	1.87 ± 0.24	1.78 ± 0.17	1.98 ± 0.28
OSI values [(*μ*mol H_2_O_2 _equivalent/L)/*μ*mol Trolox equivalent/L) × 100]			
Preoperative	0.54 ± 0.39	0.45 ± 0.26	0.61 ± 0.49
AC 20 min	0.48 ± 0.17	0.49 ± 0.22	0.47 ± 0.12^f^
Rp 30 min	0.70 ± 0.36^b^	0.57 ± 0.19	0.83 ± 0.44^g^
Rp 6 h	0.61 ± 0.32	0.50 ± 0.18	0.70 ± 0.40
Rp 48 h	0.46 ± 0.13	0.44 ± 0.09	0.49 ± 0.17

^a^When compared to preoperative value *P* = 0.045.

^b^When compared to preoperative value *P* = 0.015.

^c^When compared to preoperative value *P* = 0.047.

^d^When compared to preoperative value *P* = 0.023.

^e^When compared to preoperative value *P* = 0.037.

^f^When compared to preoperative value *P* = 0.017.

^g^When compared to preoperative value *P* = 0.048.
